# Predictors of impaired pulmonary function in people living with HIV in an urban African setting

**DOI:** 10.4102/sajhivmed.v22i1.1252

**Published:** 2021-08-17

**Authors:** Sarah E. van Riel, Kerstin Klipstein-Grobusch, Roos E. Barth, Diederick E. Grobbee, Charles Feldman, Erica Shaddock, Sarah L. Stacey, Willem D. F. Venter, Alinda G. Vos

**Affiliations:** 1 Julius Global Health, Julius Center for Health Sciences and Primary Care, University Medical Center Utrecht, Utrecht University, Utrecht, the Netherlands; 2 Division of Epidemiology and Biostatistics, Faculty of Health Sciences, University of the Witwatersrand, Johannesburg, South Africa; 3 Department of Internal Medicine and Infectious Diseases, University Medical Center Utrecht, Utrecht University, Utrecht, the Netherlands; 4 Department of Internal Medicine, Faculty of Health Sciences, University of the Witwatersrand, Johannesburg, South Africa; 5 Division of Pulmonology, Department of Internal Medicine, Charlotte Maxeke Johannesburg Academic Hospital, Johannesburg, South Africa; 6 Department of Infectious Diseases, Faculty of Health Sciences, University of the Witwatersrand, Johannesburg, South Africa; 7 Ezintsha, Wits Reproductive Health and HIV Institute, Faculty of Health Sciences, University of the Witwatersrand, Johannesburg, South Africa

**Keywords:** obstructive lung disease, COPD, asthma, HIV, sub-Saharan Africa, predictors

## Abstract

**Background:**

Studies have associated HIV with an increased risk of obstructive lung disease (OLD).

**Objectives:**

We aimed to identify the predictive factors for impaired lung function in an urban, African, HIV-positive population.

**Method:**

A cross-sectional study was performed in Johannesburg, South Africa, from July 2016 to November 2017. A questionnaire was administered and pre- and post-bronchodilator spirometry conducted. The predictors investigated included age, sex, antiretroviral treatment (ART) duration, body mass index, history of tuberculosis (TB) or pneumonia, occupational exposure, environmental exposure, smoking and symptoms of OLD (cough, wheeze, mucus and dyspnoea). Impaired lung function was defined as a forced expiratory volume in 1 second/forced vital capacity (FEV1/FVC) ratio of < 0.70, or below the 20th percentile of normal.

**Results:**

The 98 ART-naïve participants (mean age = 34.0, standard deviation [s.d.] = 8.2), 85 participants on first-line ART (mean age = 36.9, s.d. = 6.6) and 189 participants on second-line ART (mean age = 43.5, s.d. = 7.9) were predominantly female (65.6%). Of the participants, 64 (17.2%) had impaired lung function and 308 had normal lung function. Linear regression identified age (β = –0.003, *P* < 0.01), male sex (β = –0.016, *P* = 0.03) and history of TB or pneumonia (β = –0.024, *P* < 0.01) as independent predictors of a lower FEV1/FVC ratio. Following logistic regression, only a history of TB or pneumonia (odds ratio = 2.58, 95% confidence interval = 1.47–4.52) was significantly related to impaired lung function (area under the receiver operating characteristic curve = 0.64).

**Conclusion:**

Our data show that a history of TB or pneumonia predicts impaired lung function. In order to improve timely access to spirometry, clinicians should be alert to the possibility of impaired lung function in people with a history of TB or pneumonia.

## Background

In 2018, there were an estimated 37.9 million persons living with HIV (PLWH), of whom approximately 70% resided in sub-Saharan Africa (SSA).^1^ The coverage of antiretroviral treatment (ART), resulting in viral suppression, has increased in the last decades.^2,3,4^ Currently, ART coverage in adults with HIV in South Africa is 79%.^1^ Consequently, the life expectancy of PLWH has increased, and HIV is becoming a chronic disease with an increased risk of age-related comorbidities.^2,3,4^

One of the comorbid complications noted among ART-compliant HIV patients is reduced pulmonary function, including obstructive lung disease (OLD), which comprises asthma and chronic obstructive pulmonary disease (COPD).^5,6,7,8^ While HIV was found to be independently associated with COPD, the relationship between HIV and asthma remains ambiguous.^5,9,10^ The burden of these chronic diseases is high and increasing; a review by Drummond and Kirk indicates an OLD prevalence of 16% – 20% in PLWH.^11^ The underlying biological mechanisms are not yet entirely understood. Direct virus-related pulmonary toxicity, persistent systemic inflammation, a modified antioxidant/oxidant balance resulting in oxidative stress, and expedited immune deterioration are among the complex mechanisms that may explain the increased risk of OLD.^12,13^ Even in patients who achieve viral suppression on ART, these mechanisms may still continue, or the damage that occurred during uncontrolled HIV viraemia may be irreversible.^12,13^

Symptoms of OLD include cough, wheezing, dyspnoea and mucus production.^5,6,7,8,13,14^ These symptoms are often reported to be worse in HIV-positive individuals as compared to HIV-negative individuals.^5,6,7,8,13,14^ Furthermore, OLD onset is commonly earlier in this population as opposed to the HIV-negative population.^15^ A recent study suggests potential higher mortality resulting from airflow obstruction in COPD patients living with HIV compared to those without HIV infection.^3^ Undetected OLD may result in more frequent and serious exacerbations.^16,17^ Additionally, it increases the risk of comorbidities such as lower respiratory tract infections.^16,17^

In general, the failure to diagnose OLD prevents timely treatment and impacts quality of life and mortality.^16,17^ Obtaining reliable spirometry results requires adequate clinical resources and is time-consuming.^18^ Screening the vast number of HIV patients in SSA is impossible in often-overburdened healthcare systems. Hence, we aimed to identify the predictive factors for impaired lung function specific to the HIV-positive population in SSA, to allow the appropriate selection of HIV patients who would benefit most from screening for OLD by means of spirometry.

## Method

### Study population

The current study and analyses were embedded in a cross-sectional study situated at the Charlotte Maxeke Johannesburg Academic Hospital, Johannesburg, South Africa, and were undertaken between July 2016 and November 2017. The study included 394 HIV-positive individuals who were recruited in the inner city of Johannesburg. They were originally enrolled in randomised controlled trials (RCTs) comparing ART combinations in the general HIV-positive population. Recruitment from these RCTs resulted in three groups: (1) HIV-positive participants not yet on ART; (2) HIV-positive participants on first-line ART; and (3) HIV-positive participants on second-line ART. All participants had to be at least 18 years old, sign written informed consent, and understand the nature and purpose of the study. General exclusion criteria included current pregnancy, inability to undergo the study procedures (i.e. complete inability to undergo spirometry testing for any reason, such as facial anomalies), unknown HIV status, impaired kidney or liver function, and hepatitis B infection.

Participants in the ART-naïve group were recruited from an ongoing RCT comparing two novel first-line ART combinations.^19^ The enrolment period lasted from February 2017 until May 2018.^19^ All participants attending a follow-up visit between February 2017 and November 2017 were eligible for our study. To be enrolled in this group, a maximum of 8 weeks on ART was allowed.

Additional participants for the first-line ART group were enrolled from a trial comparing two older first-line ART regimens.^20^ The RCT was completed in 2016. To be included in the current study, participants had to have been on a tenofovir-containing treatment for at least 2.5 years or exceeded the 8-week ART cut-off of the ART-naïve group.

Lastly, the participants on second-line ART were recruited from a non-inferiority RCT comparing ritonavir-boosted darunavir with ritonavir-boosted lopinavir.^21^ This trial took place from June 2016 until June 2017 and included adult HIV-1 patients on a ritonavir-boosted lopinavir second-line ART for at least 6 months.^21^ To be included in the current study, participants had to attend regular follow-up visits at any time after enrolment between September 2016 and November 2017.^19^

The recruitment of participants from the ongoing RCTs was dependent on daily logistics such as researcher availability and waiting time. No prior selection of participants was done. Participants from the RCT that was completed in 2016 (participants on stable first-line ART) were approached by phone in a random order until the required number was met.

### Impaired lung function

Impaired lung function was measured through spirometry with pre- and post-bronchodilator measurements. A handheld spirometer (CareFusion 2009) and Spida 5 software were used to determine the forced expiratory volume in 1 second (FEV1), the forced vital capacity (FVC) and the ratio between these two outcomes (FEV1/FVC ratio).^22^ The procedure was executed according to the guidelines of the American Thoracic Society (ATS) and the European Respiratory Society, requiring three acceptable and repeatable blows of maximum inspiration and effort, with a good start and smooth, continuous exhalation.^22^

Pulmonary data that did not meet the ATS criteria were considered to be insufficient and were excluded from analyses. However, if there were three post-bronchodilator curves available fulfilling the ATS criteria and only two pre-bronchodilator curves, the tests were included if the pre- and post-curves were comparable (within 150 mL difference for FEV1 and FVC), because a diagnosis of asthma was ruled out in such cases.

The Global Initiative for Asthma (GINA) defines asthma in adults as an increase in FEV1 of more than 12% and 200 mL when comparing the pre- and post-bronchodilator curves.^23,24,25^ A diagnosis of COPD was based on a post-bronchodilator FEV1/FVC ratio below 0.7 or below the 5th percentile of the predicted value (the lower limit of normal [LLN]).^26,27^ Because of the small number of OLD cases according to the Global Initiative for Chronic Obstructive Lung Disease (GOLD) and LLN criteria, in the current analyses we used ‘impaired lung function’ as an outcome, which was defined as a FEV1/FVC ratio below the 20th percentile, COPD or asthma. Of note, we will use ‘impaired lung function’ throughout the article when we refer to the definition based on the 20th percentile and ‘OLD’ when we refer to the common definition used in the literature based on the 5th percentile. Considering the absence of an accepted reference for a SSA population, the equation for ‘Afro-American’ from the Global Lung Function Initiative was used to determine the LLN.^27^

The outcome assessment was not blinded, because of the clear cut-off values for diagnosis. Hence, there was no expected influence on the results or over-diagnosis.

### Predictors

The predictor selection was based on evidence from the literature and availability in the data set.^16,28,29,30,31^ Data on the demographics, medical history and disease characteristics were collected through a standardised questionnaire, based on the World Health Organization STEPs questionnaire, World Health Survey, Medical Research Council (MRC) breathlessness scale, ATS-DLD-78-A and British MRC Respiratory Questionnaire.^32,33,34,35,36,37^ The following predictors were included: age (in years), sex, ART duration (in years), medical history of lung diseases (current or previous tuberculosis [TB] or pneumonia), occupational exposure (a job for at least one year involving dust, mining, chemicals, fumes or gases), environmental exposure (exposure to second-hand smoking inside the house during childhood or currently, or exposure to an open wood fire without a chimney for heating or cooking), smoking behaviour (never, past or current) and at least one symptom corresponding to OLD (a cough for more than two months, mucus on most days, dyspnoea [MRC ≥ 2] or at least one attack of wheezing in the past 12 months). Body mass index (BMI) was calculated through the measurements obtained during physical examination according to the formula kg/m^2^. The predictor data were obtained before the outcome data, hence blinding of the predictor assessment for the outcome was achieved. The predictor assessment was not blinded for other predictor data.

### Data analysis

The descriptive statistics were presented as the mean with standard deviation (s.d.) for normally distributed continuous data, and the categorical variables were displayed using frequencies and percentages. We performed complete case analyses, as the number of missing data was very low for all predictors and outcomes (< 5%).

The predictors were analysed in a multivariable linear and multivariable logistic regression using the FEV1/FVC ratio and impaired lung function as the respective outcomes.^23,24,25,26,27^ The predictors age, sex and BMI were included in the multivariable analyses regardless of their univariable *P*-values, because of their established association with pulmonary function.^16,26^

Other candidate predictors were selected based on a *P*-value of < 0.2 in the univariable analysis. Subsequently, the identified predictors were included in the multivariable analyses and model reduction using a stepwise backward approach with a cut-off *P*-value of 0.2. A *P*-value of 0.2 was chosen to prevent missing out on possible important predictors. Two-sided *P*-values of < 0.05 were considered statistically significant.

A receiver operating characteristic (ROC) curve and area under the ROC curve (AUC) were used to determine the logistic model’s discrimination ability. Calibration of the model was assessed through a Hosmer–Lemeshow test. A non-significant outcome of this test indicates good calibration. Bootstrapping (sample *n* = 200) was performed to correct for overfitting of the ROC curve and coefficients.

### Ethical considerations

Participants provided written informed consent. Ethical approval was obtained from the Human Research Ethics Committee of the University of the Witwatersrand (HREC number M160131).

## Results

All participants were of black African descent and consisted of 98 ART-naïve participants, 85 participants on first-line ART and 189 participants on second-line ART (Table 1). Of the participants, 64 (17.2%) had impaired lung function. Of the 64 impaired lung function cases, 46 had an FEV1/FVC ratio below the 20th percentile, which included 13 COPD cases according to the GOLD and LLN 5th-percentile cut-offs and 10 cases who also met the GINA criteria for asthma. The remaining 18 cases were found solely to have asthma, as they did not have a reduced FEV1/FVC ratio below the 20th percentile and did not meet the criteria for COPD. There was no difference in age or sex between participants with or without impaired pulmonary function (*P* > 0.1 for both comparisons). Most impaired lung function was found in participants who were on second-line ART (62.5%). Dyspnoea was the most common symptom for both the patient group with normal lung function and those with impaired lung function (23.4% and 28.1%, respectively; *P* = 0.40). Other symptoms were relatively uncommon.

**TABLE 1:** Characteristics of the study population.

Variable	No impaired lung function				Impaired lung function			
	*n*	%	Mean	s.d.	*n*	%	Mean	s.d.
No. of participants (*N* = 372)	308	82.8	-	-	64	17.2	-	-
**Patient characteristics**								
Male	111	36.0	-	-	17	26.6	-	-
Age (years)	-	-	39.2	8.8	-	-	41.1	8.7
Employed (*n* = 370)	207	67.2	-	-	45	70.3	-	-
**HIV characteristics**								
Time since HIV diagnosis (years) (*n* = 392)	-	-	6.27	5.2	-	-	6.94	5.2
CD4 count (cells/mm^3^) (*n* = 349)	-	-	495.3	263.5	-	-	613.0	305.4
Time on ART (years) (*n* = 392)	-	-	4.75	4.2	-	-	5.45	4.3
**Post-bronchodilator results**								
FEV1 (L)	-	-	2.87	0.65	-	-	2.43	0.55
FVC (L)	-	-	3.37	0.77	-	-	3.24	0.69
FEV1/FVC ratio	-	-	0.85	0.05	-	-	0.75	0.08
**HIV group**								
ART naïve	85	27.6	-	-	13	20.3	-	-
First-line ART	74	24.0	-	-	11	17.2	-	-
Second-line ART	149	48.4	-	-	40	62.5	-	-
**BMI**								
Underweight	10	3.2	-	-	1	1.6	-	-
Normal weight	149	48.4	-	-	22	34.4	-	-
Overweight	68	22.1	-	-	24	37.5	-	-
Obese	81	26.3	-	-	17	26.6	-	-
**Education (*n* = 368)**								
Primary school or less	39	12.7	-	-	9	14.1	-	-
Secondary school completed	155	50.3	-	-	30	46.9	-	-
Matric completed	90	29.2	-	-	19	29.7	-	-
College or university	21	6.8	-	-	5	7.8	-	-
**Smoking (*n* = 371)**								
Never smoker	244	79.2	-	-	48	75.0	-	-
Former smoker	20	6.5	-	-	7	10.9	-	-
Current smoker	43	14.0	-	-	9	14.1	-	-
**Occupational exposure (*n* = 365)**								
Mining work for ≥ 1 year	2	0.6	-	-	1	1.6	-	-
Dusty job for ≥ 1 year	9	2.9	-	-	2	3.1	-	-
Exposure to gas, chemical fumes or pesticides in work for ≥ 1 year	6	1.9	-	-	3	4.7	-	-
**Environmental exposure**								
Second-hand smoking at baseline (*n* = 365)	48	15.6	-	-	8	12.5	-	-
Second-hand smoking during childhood (*n* = 367)	42	13.6	-	-	5	7.8	-	-
Open fire (heating) stove without chimney (*n* = 368)	3	1.0	-	-	0	0.0	-	-
**Respiratory illnesses**								
Pneumonia in the past (*n* = 369)	18	5.8	-	-	13	20.3	-	-
Tuberculosis ever (*n* = 370)	79	25.6	-	-	30	46.9	-	-
**Physical symptoms**								
Chronic cough	6	1.9	-	-	0	0.0	-	-
Bringing up mucus (*n* = 368)	11	3.6	-	-	3	4.7	-	-
Wheezing (*n* = 390)	5	1.6	-	-	2	3.1	-	-
MRC scale of ≥ 2 (*n* = 369)	72	23.4	-	-	18	28.1	-	-

Note: Underweight, BMI < 18.5; normal weight, BMI = 18.5–24.99; overweight, BMI = 25.0–29.99; obese, BMI ≥ 30.

ART, antiretroviral therapy; BMI, body mass index; MRC, Medical Research Council; s.d., standard deviation; FEV1, forced expiratory volume in 1 second; FVC, forced vital capacity.

**FIGURE 1:**
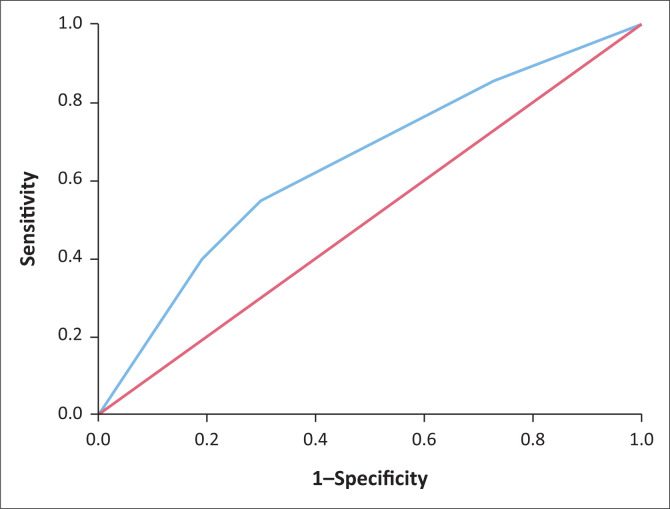
Receiver operating characteristic curve.

Following the linear regression analysis, three variables were identified as predictors of a lower FEV1/FVC ratio: age (β = –0.003, *P* < 0.01), male sex (β = –0.016, *P* = 0.03) and history of TB or pneumonia (β = –0.024, *P* < 0.01) (Table 2). The logistic regression analyses, after bootstrapping, included female sex and a history of TB or pneumonia as predictors of impaired lung function (odds ratio for female sex = 1.44, 95% confidence interval [CI] = 0.78–2.66; odds ratio for history of lung disease = 2.58, 95% CI = 1.47–4.52). Only a history of TB or of pneumonia remained a statistically significant predictor (*P* < 0.001) (Table 3). The logistic regression analyses resulted in the following diagnostic model: probability of impaired lung function = 1/(1 + exp (–2.24 + 0.37 × female sex + 1.01 × history of pneumonia or TB). The ROC curve is presented in Figure 1. After bootstrapping, the AUC was 0.64 (95% CI = 0.56–0.70), which indicates that the prediction rule is able to identify participants with impaired lung function at an acceptable level, although not very well. The Hosmer–Lemeshow test yielded a *P*-value of 0.97.

**TABLE 2:** Univariable and multivariable associations with the ratio of forced expiratory volume in 1 second to forced vital capacity.

Variable	Univariable			Multivariable		
	β	s.e.	*P*	β	s.e.	*P*
Constant	-	-	-	0.966	-	-
Age (years)	−0.003	0.00	**< 0.01**	−0.003	0.00	**< 0.01**
Sex (male)	−0.017	0.01	**0.02**	−0.016	0.01	**0.03**
BMI	−0.001	0.00	**0.25**	−0.000	0.00	0.64
Environmental exposure	0.004	0.01	0.62	-	-	-
Occupational exposure	0.007	0.02	0.63	-	-	-
History of lung disease (TB, pneumonia)	−0.029	0.01	**< 0.01**	−0.024	0.01	**< 0.01**
ART duration (years)	−0.003	0.00	**< 0.01**	0.002	0.00	0.07
At least one OLD symptom	−0.019	0.01	**0.01**	−0.012	0.01	0.10
**Smoking status**						
Never	0.000	-	-	-	-	-
Past	−0.012	0.01	0.37	-	-	-
Current	−0.007	0.01	0.43	-	-	-

Note: *N* = 372. Variables with *P*-values in bold in the univariable regression were considered for multivariable regression; those in bold for the multivariable regression were considered statistically significant.

TB, tuberculosis; ART, antiretroviral treatment; BMI, body mass index; OLD, obstructive lung disease; s.e., standard error.

**TABLE 3:** Univariable and multivariable associations with impaired lung function.

Variable	Univariable			Multivariable		
	Odds ratio	95% CI	*P*	Odds ratio	95% CI	*P*
Constant	-	-	-	0.11	-	-
Age (years)	1.03	0.99–1.06	**0.11**	-	-	-
Sex (female)	1.56	0.85–2.84	**0.15**	1.44	0.78–2.66	0.24
BMI	1.03	0.99–1.08	**0.16**	-	-	-
Environmental exposure	0.71	0.35–1.42	0.33	-	-	-
Occupational exposure	0.84	0.24–2.96	0.79	-	-	-
History of lung disease (TB, pneumonia)	2.81	1.62–4.87	**< 0.01**	2.74	1.58–4.76	**< 0.01**
ART duration (months)	1.04	0.98–1.11	0.23	-	-	-
At least one OLD symptom	1.30	0.73–2.32	0.37	-	-	-
**Smoking status**						
Never (reference)	1.00	-	-	-	-	-
Past	0.94	0.43–2.06	0.88	-	-	-
Current	1.67	0.55–5.13	0.37	-	-	-

Note: The multivariable figures are estimated after internal validation and shrinkage (*n* = 372; number of events = 64). Variables with P-values in bold in the univariable regression were considered for multivariable regression; those in bold for the multivariable regression were considered statistically significant.

95% CI, 95% confidence interval; TB, tuberculosis; BMI, body mass index; ART, antiretroviral treatment; OLD, obstructive lung disease.

## Discussion

We have explored and internally validated, by means of bootstrapping, the predictors of impaired lung function in an HIV-positive population situated in urban SSA. Combining the results from the linear and logistic regressions suggested that a history of TB or pneumonia predicts impaired lung function in the current population. Male sex and increasing age were also associated with a decrease in lung function in the linear regression analyses.

Existing models of OLD focus on the added values of other diagnostic tools, such as different types of computed tomography and magnetic resonance imaging, or of capnography to enable an earlier or more reliable diagnosis.^38,39,40,41^ Other models aim to predict the prognosis or exacerbations of OLD.^38,39,40,41^ However, none of these models focused on the diagnostic value of easily collected predictors, and none of the models was developed in an HIV-positive, SSA population. Our diagnostic model has only modest capacity to distinguish between normal lung function and impaired lung function. Hence, as our model is the first to use readily available clinical data, it establishes the foundation for further development of a diagnostic model of OLD in the current population.

Our findings on individual predictors are in line with previous research that has been conducted in HIV-positive populations in diverse countries.^9,28,29,30,31,42,43,44,45,46,47^ Increasing age, a history of TB, and prior bacterial or *Pneumocystis* pneumonia have been associated with COPD, asthma and lower FEV1/FVC ratios in multiple studies.^9,28,29,30,42,43,44,45,46,47^ Most evidence has been generated by research conducted in high-income countries. Only a limited number of studies conducted in SSA have analysed the association between HIV and impaired lung function.^9,29,48,49,50^ The factors associated with impaired lung function in the SSA population were older age and a history of TB.^9,49,50^ Sex, viral load, smoking status and ART duration have not been conclusively identified as impaired lung function predictors.^9,29,49,50^ Furthermore, a systematic review by Finney et al. found that most of the studies conducted in SSA focus on the prevalence of impaired lung function rather than identifying the predictors.^48^ Most studies aiming to identify the predictors had a limited sample size^9,29,49^ or a low prevalence of impaired lung function,^29,50^ so the robustness of the findings has to be confirmed in other research.

A vast body of research has established male sex as a risk factor for airflow obstruction.^42,43,44,45,46,47^ This association was confirmed through our linear regression analyses. However, a significant association between sex and impaired lung function was not found in the logistic regression. This is likely the result of limited statistical power in the dichotomous analysis. Another possible explanation is the inclusion of asthma cases in the impaired lung function outcome. Previous studies conducted in HIV-positive populations have found asthma patients to be more frequently female.^30,51^ The asthma cases were not included in the linear regression, as these analyses focused on the FEV1/FVC ratio. Hence, the influence of male sex on the impaired lung function outcome may have been counterbalanced by the association between female sex and asthma.

Strikingly, smoking was not related to impaired lung function, while a vast body of previous research has established smoking as a strong predictor of and risk factor for OLD in PLWH.^44,45,52,53,54^ Nevertheless, two studies analysing HIV-infected populations have found similar results as those currently reported.^9,55^ This may be explained by inconsistent smoking behaviour as a result of insufficient finances.^56,57^ Furthermore, smoking behaviour is usually assessed through inhaled smoking, while in the current population, chewing tobacco and the use of snuff are common as well.^56,57^ Hence, there may be unmeasured smoking behaviour that is not being taken into account. Furthermore, it has been suggested that the pathogenesis of OLD may be independent of smoking behaviour in the HIV-positive population, attributable to a state of chronic inflammation, but evidence is lacking.^58^

The current study has not identified ART duration as a predictive factor of impaired lung function. Research has not reached a consensus regarding the effect of ART on OLD.^44,45^ Some evidence has identified ART as an independent factor for airway obstruction in HIV patients.^44,45^ The underlying biological mechanism may be related to immune reconstruction inflammatory syndrome or the initiation of pulmonary autoimmunity.^58^ Other studies have found no significant effect of ART on OLD. Reasons for these conflicting findings may be related to the HIV stage and time of ART initiation, as well as differences in the study population.^30,59^

This study has several strengths. Our model is based on comprehensive and detailed data from an HIV-positive population in an urban SSA setting and is the first to use readily available clinical data. Missing data were limited, and standardised methods of assessment were used to obtain the predictors and outcome data. Our study responds to the need for identifying the predictive factors for impaired lung function as called for in recent articles.^9,31,58^ Limitations include that the current data set had too few COPD and asthma cases according to the GINA, GOLD and LLN criteria to develop a model for OLD. Hence, we used a FEV1/FVC ratio below the 20th percentile, which inherently leads to over-diagnosis of clinical OLD and results in limited generalisability. A larger data set with more clinical OLD cases would allow for verification of the identified predictors. The AUC indicates that the current model misses other important predictors that are necessary to improve the model performance. Assessment of smoking may have misrepresented current smoking behaviour, as no information on chewing tobacco was collected. Additionally, the study population was recruited from RCTs and might therefore not represent the general HIV-positive population in Johannesburg. However, we do not foresee bias in the current study, as RCT participation is attractive to the general population in Johannesburg because of the convenient logistics (no long queues) and the quality of medical services involved.

## Conclusion

A history of TB or pneumonia predicted impaired lung function in the current population. Male sex and increasing age in years were associated with a decrease in lung function. The model does not yet allow adequate risk stratification to guide clinical management, but clinicians should be aware of possible impaired lung function in HIV-positive patients with a history of TB or pneumonia and consider early spirometry testing for this group. More research is needed to confirm the identified predictors and to add others in order to develop a model that is specific and sensitive enough to rule out or rule in OLD.
